# Numerical and Experimental Study of Microchannel Performance on Flow Maldistribution

**DOI:** 10.3390/mi11030323

**Published:** 2020-03-20

**Authors:** Jojomon Joseph, Danish Rehman, Michel Delanaye, Gian Luca Morini, Rabia Nacereddine, Jan G. Korvink, Juergen J. Brandner

**Affiliations:** 1 MITIS SA, Rue del Rodje Cinse 98, 4102 Seraing, Belgium; michel.delanaye@mitis.be (M.D.); rabia.nacereddine@mitis.be (R.N.); 2 Institute of Microstructure Technology, Karlsruhe Institute for Technology, 76131 Karlsruhe, Germany; jan.korvink@kit.edu (J.G.K.); juergen.brandner@kit.edu (J.J.B.); 3 Microfluidics Laboratory, Department of Industrial Engineering (DIN), University of Bologna, Via del Lazzaretto 15/5, 40131 Bologna BO, Italy; danish.rehman2@unibo.it (D.R.); gianluca.morini3@unibo.it (G.L.M.)

**Keywords:** micro channel, reduced model, wire-net perturbators, s-shaped perturbators, high-temperature heat exchangers

## Abstract

Miniaturized heat exchangers are well known for their superior heat transfer capabilities in comparison to macro-scale devices. While in standard microchannel systems the improved performance is provided by miniaturized distances and very small hydraulic diameters, another approach can also be followed, namely, the generation of local turbulences. Localized turbulence enhances the heat exchanger performance in any channel or tube, but also includes an increased pressure loss. Shifting the critical Reynolds number to a lower value by introducing perturbators controls pressure losses and improves thermal efficiency to a considerable extent. The objective of this paper is to investigate in detail collector performance based on reduced-order modelling and validate the numerical model based on experimental observations of flow maldistribution and pressure losses. Two different types of perturbators, Wire-net and S-shape, were analyzed. For the former, a metallic wire mesh was inserted in the flow passages (hot and cold gas flow) to ensure stiffness and enhance microchannel efficiency. The wire-net perturbators were replaced using an S-shaped perturbator model for a comparative study in the second case mentioned above. An optimum mass flow rate could be found when the thermal efficiency reaches a maximum. Investigation of collectors with different microchannel configurations (s-shaped, wire-net and plane channels) showed that mass flow rate deviation decreases with an increase in microchannel resistance. The recirculation zones in the cylindrical collectors also changed the maldistribution pattern. From experiments, it could be observed that microchannels with S-shaped perturbators shifted the onset of turbulent transition to lower Reynolds number values. Experimental studies on pressure losses showed that the pressure losses obtained from numerical studies were in good agreement with the experiments (<4%).

## 1. Introduction

In recent years, Computational Fluid Dynamics (CFD) has been widely used to analyze heat exchanger performance and optimize it for specific applications. In the micro-gas turbine (MGT) industry, it is challenging to maintain laminar flow in microchannels. On the other hand, it is well-known that perturbators lead to local turbulences and, therefore, increase the heat transfer performance. Min et al. [1] presented different heat exchanger design schemes for gas turbine applications. They suggested that heat transfer effectiveness must be higher than 90% and pressure losses must be less than 3%, along with excellent resistance to oxidation and creep for temperatures above 650 °C. Based on a recent market study on MGT for decentralized energy systems made by Xiao [2], the target for thermal effectiveness must be higher than 90% and pressure losses must be less than 5%. Shah [3], in his review paper, noted similar performance requirements. 

Microchannel entrance length effects, as well as flow maldistribution [4], plays an essential role in compact heat exchanger performance. The temperature distribution effect on the flow maldistribution for a parallel microchannel system was meticulously investigated by Siva [5]. He figured out that a high heat flux or highly heated areas induced a reduction in viscosity of fluid, resulting in a higher flow maldistribution. Different types of collectors were analyzed by Siddique [6], and it was found that the creation of a stagnation zone, the growth of a boundary layer along the collector wall and low/high-velocity zones in the collector were the prime causes of flow maldistribution.

Microchannel performance plays a vital role in heat exchanger assembly. Tao [7] suggested heat transfer enhancement by decreasing the thermal boundary layer, introducing protrusions inside the microchannels and increasing the velocity gradient near the heated surface. Transverse vortex generators and longitudinal vortex generators play a significant role in local and global heat transfer enhancements, as shown in [8]. Valencia [9] showed that at Reynolds number 2000 heat transfer was enhanced by 30% due to turbulence together with a five-fold increase in friction factor. Increasing the blockage ratio and reducing the aspect ratio increased the global Nusselt number. An analysis by Paolo [10] of Conjugate Heat Transfer (CHT) on a cube revealed that the heat transfer was minimum when the boundary layer was undisturbed.

Smooth pipes (with smaller roughness) have friction factor behavior similar to the conventional theory. On the contrary, rough tubes have showed an earlier turbulent transition (Re = 350) [11,12,13,14]. Thus, adding perturbators in microchannels can induce turbulent transitions at smaller Reynolds numbers. At the same time, near-wall turbulence causes a significant share of pressure losses. Increasing the area density will increase the pressure losses and result in an increased fouling effect. Studies have showed that a high level of turbulence intensifies the self-cleaning inside complex microchannels [15]. On the contrary, increasing size to overcome pressure losses results in higher capital and reduces the performance advantages of miniaturization.

Yang [16,17] compared the performance of heat exchange devices with different microstructures manufactured on thin foils to enhance heat transfer. He found that “partition foils having low thermal conductivity can enhance the heat transfer by decreasing the axial conduction” and that, at a microscale, cross-flow configuration tends to provide similar results to the counter-current arrangement at a large Reynolds number (>2400). This is because, even if the arrangement is counter-current, there is always a cross-flow part in microchannel arrangements due to the microchannels with plane partition foils [17]. This fact led to the wire-net arrangement in our study, which was created to suppress the cross-flow pattern in the counter-flow channels.

Microchannel performances of wire-net and S-shape perturbators were presented by Joseph [18]. Joseph [18,19,20,21] also proposed a Reduced Order Model (ROM) to investigate the collector performance. The global parameters of the heat exchanger (overall thermal efficiency and pressure losses) were investigated using the ROM and were validated experimentally based on high-temperature testing of the full-scale heat exchanger. The experimental study used to validate the employed numerical model in terms of flow maldistribution, microchannel pressure loss and so on was not presented. In this paper, an experimental study is performed to investigate flow maldistribution and microchannel pressure losses based on a pressure-loss study (without temperature effects). In addition, an experimentally validated ROM was utilized to investigate the influence of collector performance on microchannel characteristics like foil thickness, type of perturbators and collector recirculation zones.

## 2. Numerical Model

A single heat exchanger block comprises of a secondary collector with 60 microchannels. The number of blocks is calculated based on the total mass flow rate, required pressure losses and thermal efficiency. A primary collector supplies and collects the mass flow rate to each block of heat exchangers. Thus, flow maldistribution in both primary and secondary collectors play a crucial role in the performance of the heat exchanger assembly. A computationally inexpensive methodology that has been presented by Joseph [20] will be utilized for a detailed investigation of collector performance. 

### 2.1. Microchannel Design

**Figure 1:**
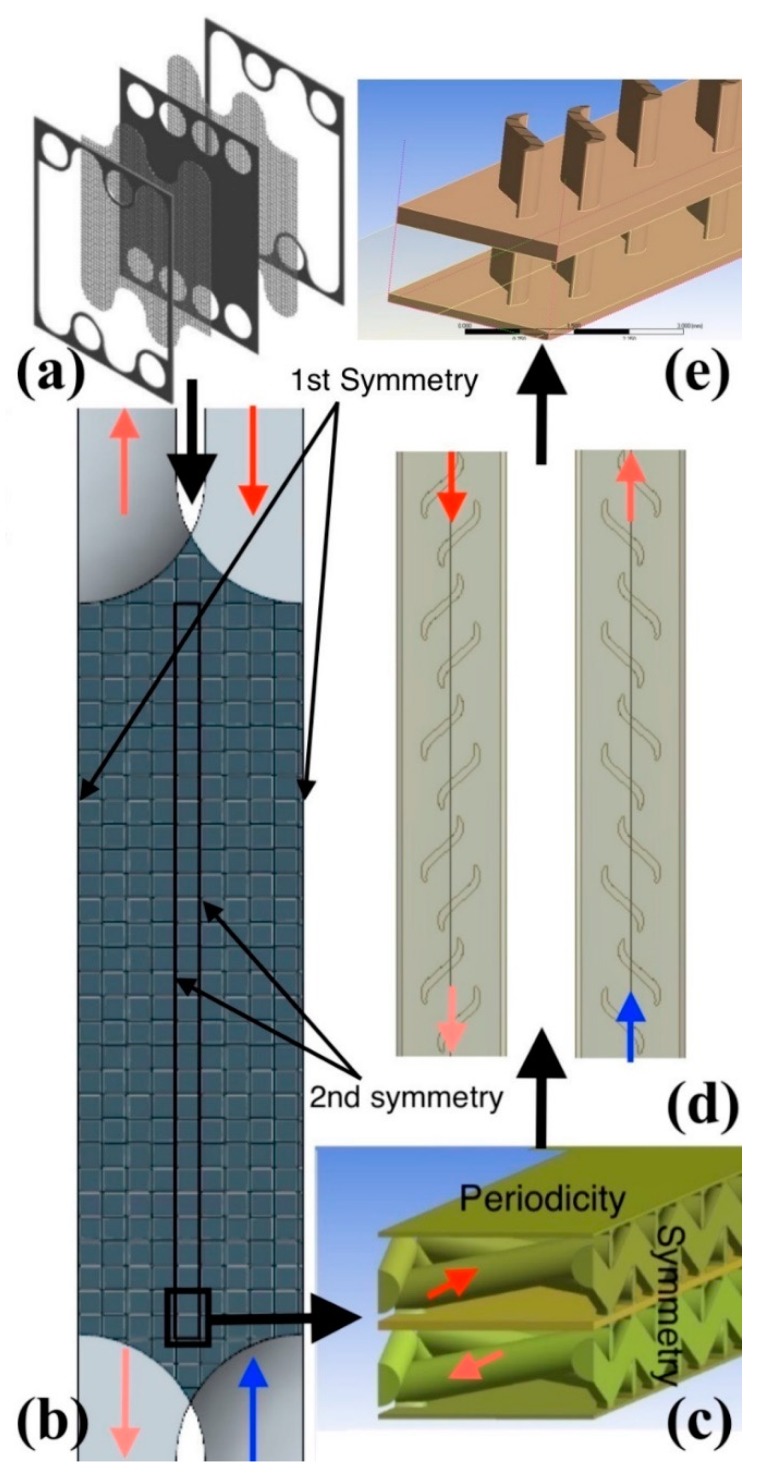
Wire-net brazed heat exchanger arrangement (**a**); microchannels with wire-net perturbators (**b**); Conjugate Heat Transfer (CHT) domain (**c**); wire-net microchannels replaced by S-shaped perturbators (**d**); CHT domain (**e**).

Two types of microchannel configurations, wire-net and S-shape perturbators, were selected for detailed microchannel CFD analysis. A comparative study was carried out by replacing the wire-net perturbators with S-shape perturbators. The wire-net heat exchanger consist of partition foils that are separated by bobbing wire-net structures. The patented wire-net heat exchanger [18,19,20,21] is assembled as a stack of counter-flow channels with optimized thicknesses separated by thin partition foils. The frames are brazed together with the partition foils (see Figure 1a). Cylindrical collectors provide cylindrical-shaped microchannel inlets that distribute mass flow rates uniformly to the wire-net microchannels. Cylindrical collectors with wire-net microchannels are depicted in Figure 1a. 

High computational effort is needed to simulate entire counter-flow wire-net microchannels. Two types of geometrical symmetry exist in a plane that is parallel to the inlet flow direction (see Figure 1a). The primary symmetrical domain (see Figure 1b) is at half of the secondary collector’s diameter, while the secondary symmetrical domain is located at the center of the wire-net compact heat exchanger (see Figure 1c). Again, high computational effort is needed to carry out a Conjugate Heat Transfer (CHT) analysis by holding the primary symmetry (see Figure 1b). The objective of the preliminary analysis based on the primary symmetrical domain is to investigate the homogeneity in the wire-net flow physics and verify the second symmetry assumption for the CHT analysis. Thus, a turbulent K-ω Reynolds averaged Navier-Stokes (NS) model without energy equations was adopted for the preliminary study to reduce solver complexity. Apparently, the second analysis based on the second symmetrical domain was performed to investigate the Conjugate Heat Transfer effects in microchannels. 

Similarly, periodic boundary conditions were implemented in the plane that is perpendicular to the inlet flow direction. A periodicity exists in half of the microchannel foil thickness on every hot and cold side alternatively. Thus, the counter-flow arrangement was simplified into single hot and cold microchannels with partition foil above, as shown in Figure 1c.

Three-dimensional, ideal gas, steady Conjugate Heat Transfer (CHT) simulations were carried out in counter-flow ducts to investigate the effect of thermal efficiency and pressure losses for various inlet mass flow rates. A turbulent K-ω shear stress transport model was utilized for all the CHT models and collectors simulations. The free microchannel simulations were performed using laminar models. The k-ε model was not accurate in the near-wall region, while the K-ω model was appropriate for near-wall turbulent flows. In the K-ω shear-stress turbulent transport model, both conventional models (K-ω & k-ε) are mixed to take advantage of their unique advantages [22].

The microchannel thermal efficiency ε [%] is calculated using the following:

(1) $$\varepsilon \left[\%\right]=\frac{C_{h}(T_{\mathrm{in}}^{h}-T_{\mathrm{out}}^{h})}{\min\left(C_{c},C_{h}\right)(T_{\mathrm{in}}^{h}-T_{\mathrm{in}}^{c})}\times 100$$

(2) $$C_{c}=m_{\mu c}{\mathrm{Cp}}_{c}\;;C_{h}=m_{\mu h}{\mathrm{Cp}}_{h}$$

where ${\mathrm{Cp}}_{c/h}$ are the hot and cold fluid capacity rates, respectively; $T_{\mathrm{in}/\mathrm{out}}^{h}$ and $T_{\mathrm{in}/\mathrm{out}}^{c}\;$are the inlet and outlet temperatures of the counter-flow channels, respectively; and $m_{\mu c/\mu h}$ are the microchannel mass flow rates at the hot and cold inlets, respectively. The microchannel pressure losses ∆P [%] were calculated using the following:

(3) $$\Delta P\;\left[\%\right]=\frac{P_{\mathrm{in}}-{\;P}_{\mathrm{out}}}{P_{\mathrm{ab}}}\times 100$$

where $P_{\mathrm{in}/\mathrm{out}}$ are the inlet and outlet absolute pressure, respectively; and $P_{\mathrm{ab}}$ is the absolute pressure at the inlet. The influence of pressure losses on density is relatively low if the total pressure loss is less than 5%. Morini [23] considered in his studies that flow is only incompressible when the Mach number is less than 0.3, or when the relative pressure loss ∆*P/*$P_{\mathrm{ab}}$ is less than 5%. In our present study, the maximum pressure loss expected was 5%. As a result, the flow regime remains incompressible, although density variations as a function of the inverse of temperature as well as the fluid were dilatable [23]. As a result, a segregated solver is adopted. 

### 2.2. Porous Medium Model 

**Figure 2:**
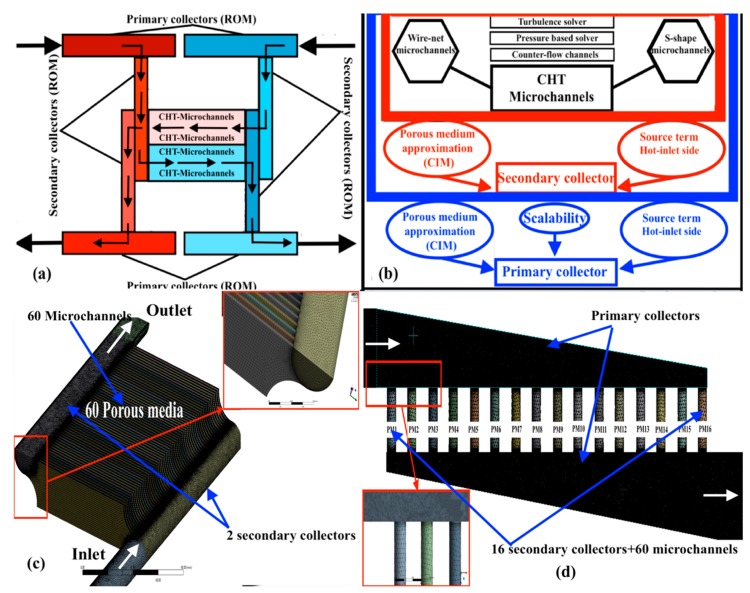
Scheme of the heat exchanger assembly (**a**); numerical methodology (**b**); secondary collector model (**c**); primary collector mesh model with 16 porous mediums replicating 16 collectors (**d**).

The CFD methodology based on a porous medium approximation was implemented to investigate the flow maldistribution. A general scheme of the heat exchanger arrangement is depicted in Figure 2a. The microchannels of the wire-net/S-shape fins have a complicated structure that again leads to high simulation needs. The best method is to split the heat exchanger configuration part by part and analyze the performance using a Reduced Order Model (ROM). 

The modelling was performed in three different stages (see Figure 2b):

1. Microchannel CHT model. Estimating the microchannel performance based on detailed three-dimensional CHT analysis.
2. Reduced model for cylindrical secondary collectors (see Figure 2c). Microchannels are replaced by porous medium models to evaluate the flow maldistribution and thereby the secondary collector performance. The microchannels have a very coarse mesh (10,000 cells per channel), and the secondary collector has a highly refined mesh to capture the secondary effects.
3. Reduced model for trapezoidal primary collectors (see Figure 2d). Scalability of the ROM was utilized to model both the microchannel along with the secondary collectors as a single porous medium. Therefore, the effects of both microchannels and secondary collectors were introduced into a cylindrical duct. The cylindrical duct has a very coarse mesh, and the primary collector has a highly refined mesh. An overall heat exchanger performance is estimated based on flow maldistribution and overall pressure losses.

In the first stage of the CFD modelling, a detailed CHT analysis was carried out for a microchannel section with specific boundary conditions. Microchannel performance characteristics were utilized to model the microchannels as a porous medium, with inertial and viscous coefficients as the main parameters.

In the second stage of modelling, the microchannels were considered a porous medium, and the cylindrical secondary collector performance was evaluated. The cylindrical cross-section of the secondary collectors was extruded at both the outlet and inlet for better convergence, as depicted in Figure 2c. The Constant Integration Method (CIM) based on the Darcy-Forchheimer law was utilized to calculate the porous medium characteristics that replicate the Conjugate Heat Transfer model. The computational domain was sharply reduced, since the microchannels were examined as a porous medium with a free-slip boundary condition.

In the third stage of modelling, the microchannels with secondary collectors were considered a porous medium, and the scalability of the ROM provided an easiness with which to create the temperature and pressure jump across circular of cross-sections. Scalability of the ROM was developed to characterize the primary collector performance and thereby the overall heat exchanger performance for the micro combined heat and power (CHP) system. 

The primary and secondary collector mesh model is depicted in Figure 2a,b, respectively. Half a section of the cylindrical collectors that feeds 60 microchannels is depicted in Figure 2a. The global performance of a secondary collector with 60 microchannels was implemented into a cylindrical duct with a trapezoidal primary collector configuration (see Figure 2b). A cylindrical channel with a porous medium approximation replaced the effects of the secondary collector and 60 microchannels. 

The number of microchannels, together with the microchannel size reduction (γ) coefficient, was utilized to calculate the microchannel inlet mass flow rate from the collector inlet mass flow rate,

(4) $$m_{\mu \mathrm{CHT}}=\frac{m_{\mathrm{co}}}{{\gamma n}_{\mathrm{pl}}}$$

where $m_{\mu \mathrm{CHT}}\;$is the microchannel mass flow rate$,\;m_{\mathrm{co}}$ is the collector mass flow rate, $n_{\mathrm{pl}}$ is the number of plates/channels and γ is the microchannel size-reduction coefficient calculated due to simplification of the computational domain (symmetric and periodic boundary conditions). The primary collector inlet mass flow rate was calculated using the following:

(5) $${\;m}_{\mathrm{co}}=\frac{m_{\mathrm{to}}}{2n_{\mathrm{bl}}}$$

where $m_{\mathrm{co}}$ is the secondary collector mass flow rate, $m_{\mathrm{to}}$ is the total mass flow rate and $n_{\mathrm{bl}}$ is the number of blocks/modules.

#### Reduced Order Model (ROM)

Global parameters such as pressure loss, thermal efficiency and turbulent viscosity were determined from a three-dimensional CHT analysis of microchannels. This was utilized to calculate the inertial and viscous coefficients (porous medium model) of the reduced model using CIM. The Darcy-Forchheimer law was modified and implemented to account for the significant temperature variation (∆T=600 K) and localized turbulence effect in the microchannels [19]:

(6) $$\begin{array}{l} \frac{\left(P_{\mathrm{in}}-P_{\mathrm{out}}\right)}{L}\\ =\frac{1}{\alpha }\frac{L}{\Delta T}\frac{m}{A}\frac{R}{P}\{{\left[T^{\frac{1}{2}}\left(3T^{2}-5S_{1}T+15S_{1}^{2}\right)-15S_{1}^{\frac{5}{2}}\arctan{\left(\frac{T}{S_{1}}\right)}^{\frac{1}{2}})+\epsilon_{m}\frac{\mathrm{PT}}{R}\right]}_{T_{0}}^{T_{1}}\\ +\frac{1}{2}C_{2}\frac{m}{A}{\left[\frac{T^{2}}{2}\right]}_{T_{0}}^{T_{1}}\} \end{array}$$

Power per unit volume was implemented as a source term along the microchannels (see Equation (7)). Joseph [19,20] applied the new approach and validated the porous medium model (with modified inertial and viscous coefficients) for secondary collectors.

(7) $$Q=m_{\mu \mathrm{CHT}}(T_{\mathrm{in}}^{h/c}-T_{\mathrm{out}}^{h/c}){\mathrm{Cp}}_{h/c}$$

The model was validated numerically and experimentally [18,19,20]. Rehman et al. [24] used a similar approach for microchannels with large density variations (compressible flows). The collector’s flow maldistribution influenced the mass flow rate and thereby the heat transfer prediction was biased along with total pressure losses.

## 3. Results and Discussion 

### 3.1. Microchannel Performance

**Figure 3:**
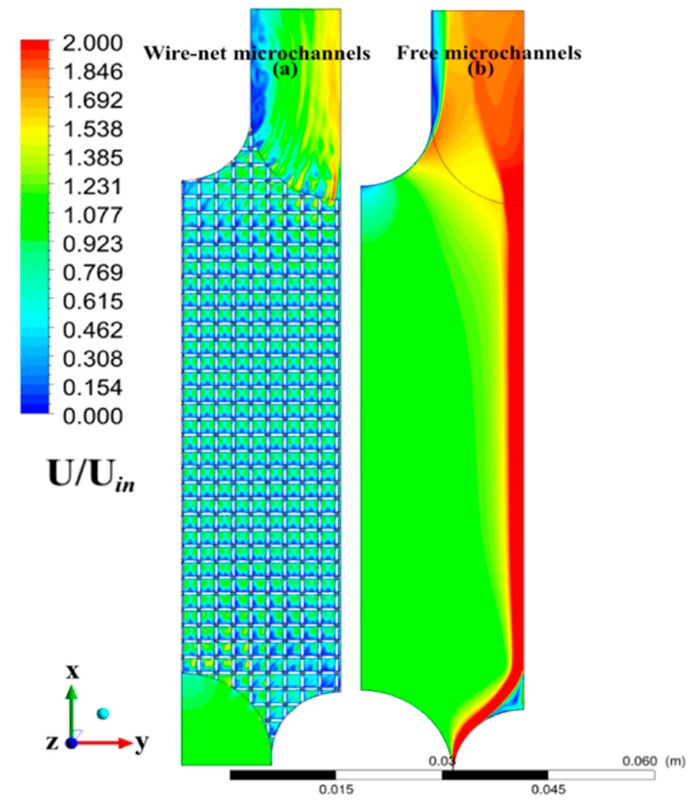
Non-dimensional velocity contours of microchannels with and without the wire-net.

The non-dimensional velocity contours (with and without the wire-net) from the preliminary analysis based on the primary symmetrical domain are depicted in Figure 3. The non-uniform velocity distribution in the microchannels (without the wire-net) adversely affect the compact heat exchanger performance, as mentioned by Yang et al. [25], due to lower resistance from the inlet to the outlet. This reduced the benefits of counter-flow passages, since the maximum velocity gradients lied at two extreme inlets (see Figure 3). However, the microchannels with the wire-net showed a more homogeneous velocity distribution that retained the counter-flow effects (see Figure 3a). These effects were similar to the conventional grid turbulence theory [26], where a grid generates turbulence that is nearly homogeneous and isotropic. Apparently, conventional grid turbulence theory is the major assumption in most of the turbulence models [27]. Thus, the numerical model can be simplified (see Figure 1c,e) for a detailed CHT analysis.

**Figure 4:**
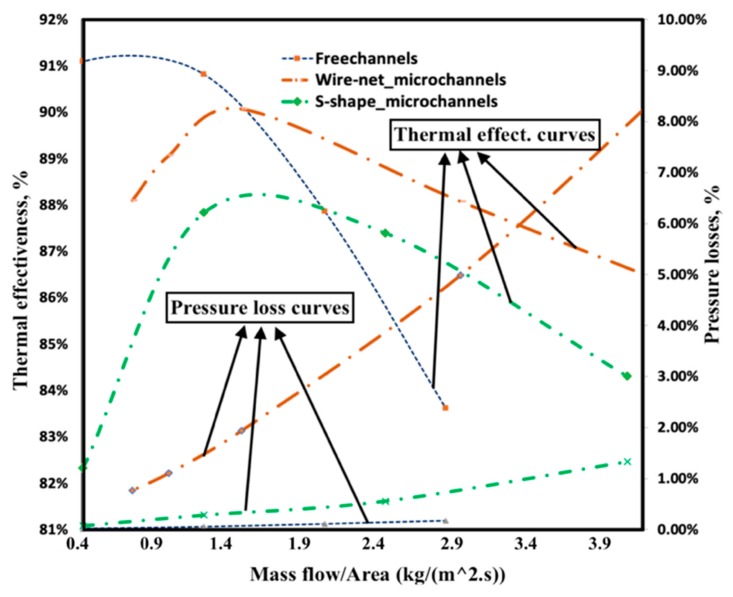
Thermal efficiency and pressure losses of free channels with the wire-net and S-shape microchannels for various mass flow rates based on CHT simulations.

CHT analysis was performed for counter-flow microchannels with symmetric boundary conditions on both sides and periodic boundary conditions of the partition foils. The acceptable thickness for metal 3D-printing is 0.25 mm. Thus, a 0.25 mm thick partition foil was selected for microchannels with S-shaped perturbators. Obviously, there is an optimum mass flow rate where the thermal efficiency reaches a maximum [19]. However, a substantial decrease in efficiency at higher mass flow rates was encountered. The steepness of this efficiency curve pattern was stronger for the S-shape perturbator than the wire-net protrusions (see Figure 4). This steepness increases the working range and thereby reduces the size and cost of the compact heat exchanger. As the steepness decreases, the microchannels can work at high mass flow rates with higher efficiency and, thereby, the number of microchannels required will be lower. As a result, heat exchangers became more compact and cost-effective. However, the pressure loss was relatively high for the wire-net at higher Reynolds numbers. The varying pressure-loss pattern (along with an increasing mass flow rate) was quadratic, which is similar to the conventional Darcy-Forchheimer law where the inertial and viscous terms are well-balanced. The free microchannels had the smallest pressure losses, as expected. The wire-net introduced higher pressure losses than the S-shaped fins (see Figure 4).

### 3.2. Collector Performance

#### 3.2.1. Secondary Collector 

##### Influence on Perturbators

**Figure 5:**
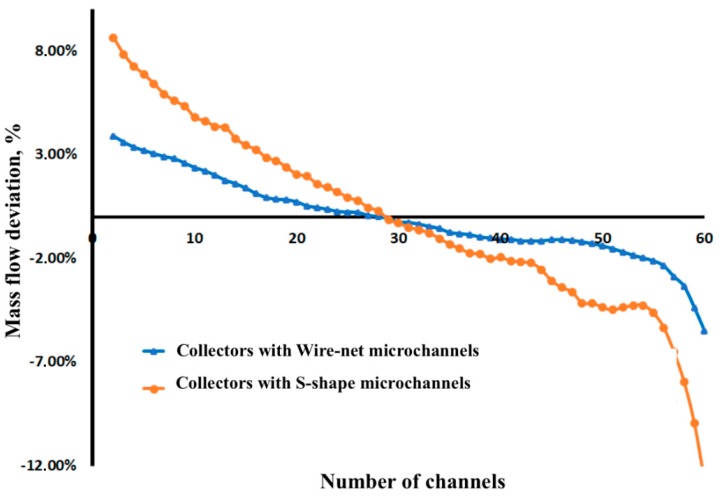
Mass flow rate deviation of perturbators based on Reduced Order Model (ROM).

The secondary collectors distributed the flow to the 60 microchannels, and the flow maldistribution is depicted in Figure 5. The collectors with lower microchannel resistance (channels with S-shape perturbators) showed a maximum deviation from the ideal CHT mass flow rate. This coincides with the studies made by Thansekhar [28] and Teng [17,29,30], which showed that the higher the flow resistance through the channels, the better the flow distribution, From these studies it can be observed that flow distribution becomes more uniform at higher flow rates, and that flow resistance increases with an increased mass flow rate. 

##### Influence on Recirculation Zones

Three different configurations were selected to investigate the collector performance:

1. microchannels without the wire-net, $m_{co}\;$= 2.76 ×10 ^−6^ kg/s;
2. microchannels with the wire-net, $m_{co}\;$= 2.76 ×10 ^−6^ kg/s; and
3. microchannels with the wire-net, $m_{co}\;$= 3.86 × 10^−6^ kg/s.

**Figure 6:**
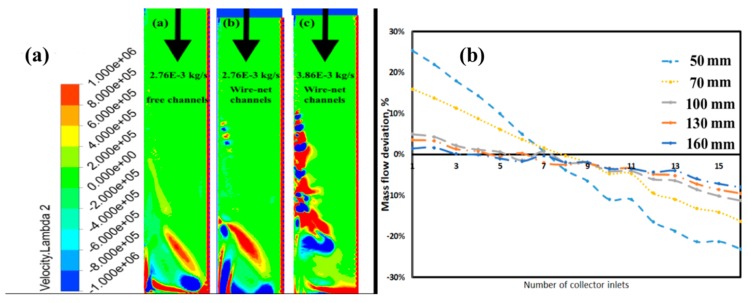
Cylindrical secondary collector performance for various microchannel pressure losses and mass flow rates (**a**); primary collector performance for different heights (**b**) based on a computational study.

The first two configurations were selected to investigate and compare the recirculation zone of cylindrical secondary collectors for different microchannel pressure losses. Further, the second and third configurations were selected to study the effect on collector inlet mass flow rates. Figure 6a shows the vorticity strength (calculated based on the Lambda 2 velocity criteria) of the recirculation zone for three different mass flow rates. The vortices originated at the beginning of the recirculation zone and remained steady enough to dissipate the integral vortex near the collector beds. 

Due to the intense mixing of the recirculating fluid with the entraining fluid, strong vortices originated along the recirculation zone. The intensity of the recirculation zones of the cylindrical collector was clear from the vorticity contour (see Figure 6a, left). Thus, the mass flow rate had a strong effect on flow maldistribution, as stated above. As microchannel pressure losses increased (see Figure 6a, center and right), vorticity strength increased. As a result, the mass flow rate distribution near the collector beds became irregular, and deviation from the CHT mass flow rate increased. In addition, the vorticity strength increased with increases in the collector inlet mass flow rate. 

#### 3.2.2. Primary Collector 

**Figure 7:**
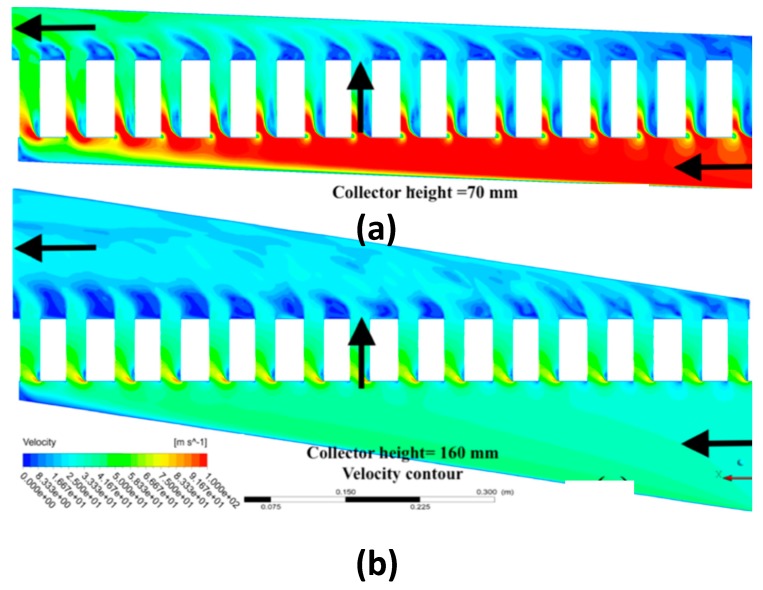
Velocity contour height = 50 mm (**a**); velocity contour height = 160 mm (**b**).

The primary trapezoidal collector inlet distributed the flow to 16 secondary collectors and then to 60 microchannels. Collector flow maldistribution strongly depended on the secondary flows in the primary collectors. A parametric study of different primary collector heights was performed, and the flow maldistribution is depicted in Figure 6b. As the collector height increased, the maldistribution decreased and the optimum collector height was found to be at 100 mm. The velocity contours of the minimum and maximum collector heights are represented in Figure 7a,b, respectively. As the height increased, the velocity near the secondary collector inlet decreased, as did the recirculation strength. The highest turbulence production for the maximum height configuration was near the primary collector outlet. The primary collector height configuration profoundly influenced the flow distribution as well as the turbulence characteristics. 

## 4. Experimental Studies

Using numerical modeling it has been established that the generation of local turbulence serves as the prime reason for enhanced heat transfer when the perturbators are used within microchannels. Such enhanced turbulence in the flow increases the friction factor, but fortunately this increase in the friction factor can easily be observed using an experimental pressure-loss analysis of adiabatic flow, without the need of a high-temperature gas flow. The thermal performance of the micro heat exchanger was numerically analyzed above using two different perturbators. Of those, this section details a microchannel with an S-shaped perturbator that puts into evidence the increased frictional losses and an earlier onset of laminar to turbulent flow transition. A complete CFD investigation of the experimental microchannel is also performed in order to validate the earlier usage of the k-ω SST turbulence model during CHT analysis to model the local turbulent flow around the perturbator. Similarly, usage of the same turbulence model in the collector studies in which microchannels are modeled as porous media can also be justified through a comparison of the experimental and numerical maldistribution patterns of the adiabatic flow. It is acknowledged that the extent of maldistribution in a diabatic operational condition would differ from that of an adiabatic case, but the selection of turbulence model can be well justified using the adiabatic case only. Therefore, trapezoidal collectors with two layers of 33 microchannels were tested experimentally using an adiabatic flow to demonstrate the close agreement between experimental and numerical evaluations of maldistribution. The details of the fabrication, test bench and experimental data reduction, along with the results of both experimental studies, are discussed in this section.

### 4.1. Microchannel 

**Figure 8:**
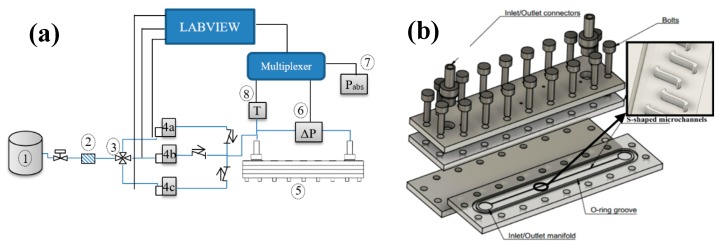
Experimental test bench (**a**) and microchannel assembly (**b**).

The test bench and apparatus for microchannels with S-shaped perturbators is depicted in Figure 8. Microchannels with S-shaped perturbators were microfabricated using a micro-milling process as shown in Figure 8b. After passing through a particle filter (2), gas stored in a high-pressure tank (1) was directed to the desired mass flow rate controller using a three way valve (3). A mass flow rate controller (4) with an operating range of 0–5000 NmL/min controlled the inlet flow. Gas was then allowed to enter the microchannel test assembly (5) and finally exit through an outer port. The total pressure loss between the inlet and the outlet of the microchannel assembly was measured utilizing a differential pressure transducer (Validyne DP15) (6) with an interchangeable sensing element that allowed accurate measurements over the whole range of encountered pressures. Atmospheric pressure was measured using an absolute pressure sensor (Validyne AP42) (7). The pressure sensor calibration was performed using a comparison test pump (Giussani BT 400) that was capable of imposing the desired pressure difference relative to the atmosphere. 

To measure the temperature at the entrance of the microchannel, a calibrated K-type thermocouple (8) was used. The thermocouple voltage and an amplified voltage of pressure sensors were fed to the internal multiplexer board of Agilent 39470A and were read through a PC using a Labview program. Uncertainties associated with all the measuring instruments are tabulated in Table 1.

The experimental friction factor was established using the following [31]:

(8) ff=(DhL)[Pin2−Pout2RTav(mΩ)2−2ln(PinPout)+2ln(TinTout)]

where L is the length, T_av_ is the average temperature of the gas between the inlet and outlet and Ω represents the cross-sectional area of the microchannel. Hydraulic diameter was defined as

(9) $$D_{h}=\frac{2\Omega }{w+h}$$

where w and h are width and height of the microchannel, respectively. Finally, Reynolds number at the microchannel inlet was defined as Re=mμDhμΩ , where μ represents the dynamic viscosity of the nitrogen gas at the inelt. Two separate theoretical correlations were used to compare the experimental friction factor for each of the laminar and turbulent flow regimes. For a fully developed flow in a rectangular channel, the laminar friction factor can be calculated using the Shah & London (S&L) correlation as [31,32]

(10) F{SL}=96(1−1.3553 β+1.9467β2−1.7012β3+0.9564β4)Re

where aspect ratio β is defined as the ratio of the depth over the width of the microchannel. The experimental friction factor was compared to the Blasius law in the turbulent regime, which is given as follows:

(11) FTurb=0.3164 Re−0.25

CFD simulations were also performed on microchannels with perturbators for the specific experimental mass flow rates. The temperature drop was negligible and not taken into account for CFD analysis. A single side of the counter-flow channels was taken for the pressure-loss studies, and 3D-Ideal gas equations were utilized using the K-ω turbulence model for CFD analysis. The microchannel length (L) with the S-shaped perturbator was 74 mm. An additional length of 9.696 mm was added to the inlet side, and 16.575 mm was added to the outlet part. This was implemented to fit the existing testbench. In conclusion, the microchannels were 101 mm in length, with 74 mm having S-shaped perturbators. 

**Figure 9:**
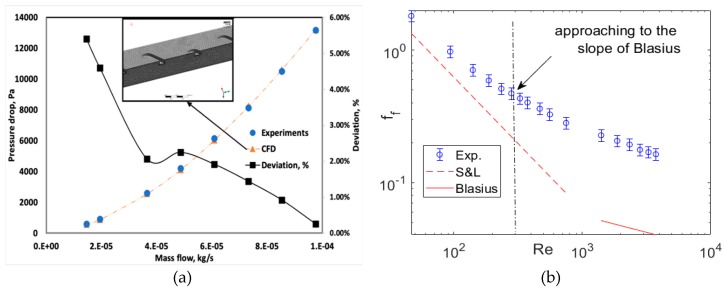
Experimental and CFD pressure-loss comparison and deviations (%) of the CFD results from the experimental pressure losses (dashed lines are for the numerical results and symbols are for the experimental readings) (**a**); experimental friction factor (**b**).

A comparison between the experimental and numerical pressure loss is shown in Figure 9a. The CFD and experimental results coincided with reasonable accuracy (<4%). The primary vertical axis denotes the pressure losses and the secondary vertical axis shows the deviation of CFD from experiments in %. The maximum deviation was larger at lower mass flow rates where the turbulent transition occurred. The RANS turbulent transition model was not reasonably accurate to predict turbulent transition. At higher mass flow rates, the CFD pressure losses were more reliable (<2%) when the flow was fully turbulent. The experimental friction factor evaluated using Equation (8) is shown in Figure 9b. The perturbators cause an increased surface roughness in the microchannels. Thus, as expected, the evaluated friction factor stayed higher than the S&L correlation in the early laminar regime. The slope of the experimental friction factor curve started deviating from the slope of laminar friction factor (S&L) and became parallel to the Blasius correlation at a considerably lower Re when compared to a smooth microchannel. The deviation in the current case began at an Re ~300, as shown in Figure 9b. This implies that the anticipated turbulent transition occurred at a lower Re, and is in agreement with several studies conducted by Shah and Wanniarachchi [11] and Heggs [12] on corrugated plate heat exchanger microchannel geometries. For Shah, the critical Reynolds number was less than 200, while Heggs [12] suggests that flow is never laminar after Re = 150. The length scale in this paper refers to the surface roughness and perturbator size in microchannels. If the length scale was too small, the turbulent transition occurred at the conventional Reynolds number. As the length scale increased, the critical Reynolds number reduced to a lower Reynolds number.

### 4.2. Collector 

**Figure 10:**
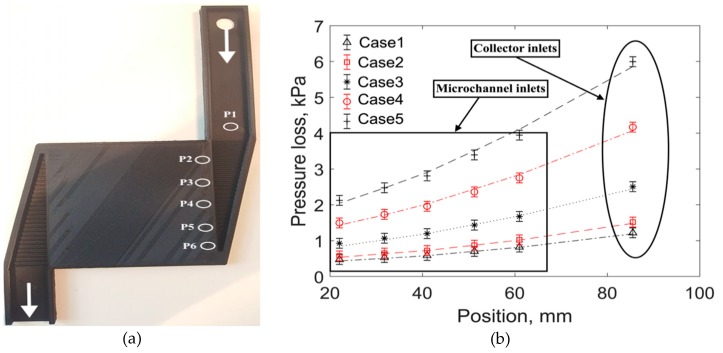
Experimental test design for collector flow maldistribution (**a**); comparison plot between experimental and CFD pressure losses (dashed lines are for the numerical results and symbols are for the experimental readings) (**b**).

A collector with a microchannel configuration was fabricated using an Ultimaker 3+ extended version 3D printer based on fused deposition modeling (FDM). An open source software known as “Cura” was used to generate the 3D printer slicing application. The 3D printer transformed the design into several cross-sections then provided successive layers, until a three-dimensional object was formed. The plastic filaments were fed into the printer and the melted plastic extruded through the nozzle head and was directed on the building plate, where it created an object layer by layer. The biodegradable polylactic acid (PLA) plastic material was selected because PLA is having additives such as color pigments, plasticizers, nucleating agents and so on to optimize the properties for the specific application. The 3D printer had two nozzles: a primary nozzle to print the body and a secondary nozzle to print supporting structures for better surface finish and quality printing. Calibration was carried out to level the building plate. The building plate is the plate in which the microchannel is extruded. The building plate was heated to 40 °C. In addition, the nozzle was heated to 210 °C. In order to avoid the adhesion issues of the first layer, normal glue was provided on the building plate. The quality of the first layer was paramount, and it was guaranteed that the first layer had nicely adhered onto the glass plate with flat lines of filament. PLA prints at moderate temperatures, specifically 210 °C, depend on the selected nozzle size and print profile. Profiles for 0.25-mm nozzles use a slightly lower temperature. A temperature of 60 °C was maintained on the building plates. An ultrasonic cleaner was used for postprocessing to remove the supporting materials. However, it was difficult to remove the supporting structures from the microchannels. Apparently, the primary nozzle AA was only used for the collector fabrication. In order to investigate the surface finish and avoid overhang structures, two layers of microchannels were fabricated separately and glued. Figure 10a shows the experimental test design without collector covering. The steps in the collectors were introduced to decrease the flow maldistribution. 

An experimental test apparatus of secondary collectors with microchannels is depicted in Figure 10a. The inlet of the secondary collectors was extruded to have a uniform flow at the collector inlets. The extruded height of the collector inlet was selected based on a CFD optimization study. Six pressure taps (P1–P6) were installed for pressure readings (see Figure 10a). Five pressure taps were installed near the microchannel inlets (P2–P6), and the remaining one (P1) was placed at the collector inlet covering. Experimental testing was carried out for five different cases. The inlet mass flow rates are shown in Table 2. The size of the pressure tap hole was 60 microns, and it was ensured that the pressure taps were placed precisely at the center of the duct.

It is relatively easy to measure the mass flow rate of selected microchannels in a heat sink or heat exchanger if liquids are used as the working fluid [28]. Moreover, optical techniques such as micro particle image velocimetry can also be employed to quantify the maldistribution of liquid flows. For a multichannel heat exchanger with gas as the working medium, however, it is challenging to measure the mass flow rate of each microchannel. Therefore, an indirect method was used in the current work where pressure loss was measured across five microchannels amongst 33 parallel microchannels. An unequal pressure profile in the trapezoidal manifold suggests an opposite profile of maldistribution, as the mass flow through each microchannel was directly linked with the associated pressure loss. Experiments were therefore carried out to investigate the collector pressure losses and compare them with CFD. The results of the comparison between CFD and the microchannel measurements are shown in Figure 10b. Microchannel inlets (P2) near the collector inlets had more pressure losses. On the contrary, the microchannel inlets (P6) that were far away from the collector inlets had lower pressure losses. The pressure-loss decreasing pattern (from the collector inlet (P1) to the last microchannel (P6)) was nearly quadratic. In addition, there was a good agreement between CFD and the experiments, and the relative error was less than 4%. This validates the employed turbulence model in the collector studies presented earlier, and therefore the maldistribution pattern predicted by the CFD model can be considered to be representative of reality.

## 5. Conclusions

The influences of wire-net and S-shape perturbators on microchannel and collector performances were evaluated separately based on a Reduced Order Model (ROM). Microchannels with a wire-net were considered for comparative studies. Furthermore, the microchannel and collector performances were experimentally analyzed based on pressure-loss studies. The wire-net microchannels showed a better performance in terms of thermal efficiency, while S-shape fins provided a better performance in terms of pressure loss. Collectors with wire-net microchannels provided a more uniform flow distribution compared to the S-shape microchannels. Collector flow maldistribution decreased with higher microchannel resistance (i.e., smaller microchannels). 

The recirculation zones in the collectors influenced the flow maldistribution. The strength of the recirculation zone near the collector beds was suppressed at higher mass flow rates. From the primary collector analysis, it was found that there was an optimum height where the pressure losses were saturated and the collector maldistribution decreased. The choice of a turbulent model was verified by comparing the CFD results with experimental tests performed for a single microchannel with S-shape perturbators and a micro heat exchanger with triangular collectors. Experimental testing of adiabatic flow showed that as a result of perturbators the onset of turbulent transition shifts to lower Reynolds number values compared to free channels. Apart from that, the microchannel and collector simulation results coincided with the experimental results with good accuracy (<4%).

## Figures and Tables

**Table 1:** Characteristics and uncertainties of the measurement instruments.

Label of Figure 8	Instrument	Range	Uncertainty
(4a)	Flowmeter (Bronkhorst EL-Flow E7000)	0–5000 (nmL/min)	± 0.6% FS
(4b)	Flowmeter (Bronkhorst EL-Flow E7000)	0–500 (nmL/min)	± 0.5% FS
(4c)	Flowmeter (Bronkhorst EL-Flow E7000)	0–50 (nmL/min)	± 0.5% FS
(6)	Differential pressure transducer(Validyne DP15)	0–35 (kPa) 0–86 (kPa) 0–220 (kPa)	± 0.5% FS
(8)	Thermocouple (K type)	0–200 (°C)	± 0.2 K

**Table 2:** Collector inlet mass flow rates for different cases.

Cases	Case 1	Case 2	Case 3	Case 4	Case 5
**Mass flow rate, kg/s**	3.01 × 10^−4^	3.36 × 10^−4^	4.32 × 10^−4^	5.54 × 10^−4^	6.66 × 10^−4^

## Abbreviations

A	cross-sectional area, m^2^
BCs	boundary conditions
CFD	computational fluid dynamics
CHP	combined heat and power
CHT	conjugate heat transfer
${\mathrm{Cp}}_{c/h}$	heat capacity at constant pressure for cold/hot inlet microchannels, J/(K.kg)
$C_{c}$	cold fluid capacity rate, W/(K)
$C_{h}$	hot fluid capacity rate, W/(K)
$D_{h}$	hydraulic diameter, mm
L	microchannel length, mm
$m_{\mu },{\;m}_{\mu c},{\;m}_{\mu h}$	microchannel mass flow rate for cold/hot inlet channels, $\frac{\mathrm{kg}}{s}$
$m_{\mathrm{co}}$	collector inlet mass flow rate for cold/hot inlet side, $\frac{\mathrm{kg}}{s}$
$m_{\mu \mathrm{CHT}}$	microchannel mass flow rate calculated from CHT model inlet, $\frac{\mathrm{kg}}{s}$
w	width of the microchannel, mm
h	height of the microchannel, mm
$n_{\mathrm{pl}}$	number of plates
PM	porous medium
$P,{\;P}_{\mathrm{in}},P_{\mathrm{out}},P_{\mathrm{ab}}$	inlet/outlet total pressure and absolute pressure, Pa
ROM	reduced order model
R	universal gas constant, $\frac{J}{\mathrm{mol}\;k}$
Re	Reynolds number
S_1_	Sutherland constant
T	temperature, K
$T_{\mathrm{in},\mathrm{out}}^{h,c}$	inlet and outlet total temperatures for hot \- cold microchannel inlets and outlets, K
T_av_	is the average temperature of the gas between the inlet and outlet, K
ΔT	temperature drop across the microchannel length, K
ΔP	pressure losses across the length, Pa
Q	thermal source term, $\frac{W}{m^{3}}$
$U,{\;U}_{\mathrm{in}}$	stream wise mean velocity, inlet velocity, $\frac{m}{s}$
1/α	viscous coefficient, $m^{-2}$
β	inertial coefficients, $m^{-1}$
β1	ratio of the depth over the width of the microchannel
Υ	microchannel size reduction coefficient
ρ	density, kg/m^3^
μ	dynamic viscosity of the Nitrogen gas, kg/(m·s)
ε	thermal efficiency, %
Є_m_	turbulent viscosity, m^2^·s^−1^
